# A novel missense mutation in the *CLCN7* gene linked to benign autosomal dominant osteopetrosis: a case series

**DOI:** 10.1186/1752-1947-7-7

**Published:** 2013-01-09

**Authors:** Ban Mousa Rashid, Nawshirwan Gafoor Rashid, Ansgar Schulz, Georgia Lahr, Beston Faiek Nore

**Affiliations:** 1 Department of Biochemistry, School of Pharmacy, Faculty of Medical Sciences, University of Sulaimani, Kurdistan Regional Government, Sulaimaniyah, Iraq; 2 Department of Hematology, Hiwa Hematology-Oncology Center, Kurdistan Regional Government, Sulaimaniyah, Iraq; 3 Department of Pediatrics and Adolescent Medicine, University Medical Center Ulm, Eythstr. 24, Ulm D-89075, Germany; 4 Department of Histopathology, Shorsh General Hospital, Kurdistan Regional Government, Sulaimaniyah, Iraq; 5 Department of Biochemistry, School of Medicine, Faculty of Medical Sciences, University of Sulaimani, Kurdistan Regional Government, Sulaimaniyah, Iraq

**Keywords:** Autosomal dominant osteopetrosis, *CLCN7* gene, Consanguineous marriage, Dominant allele, Osteopetrosis

## Abstract

**Introduction:**

Osteopetrosis is a rare inherited genetic disease characterized by sclerosis of the skeleton. The absence or malfunction of osteoclasts is found to be strongly associated with the disease evolution. Currently, four clinically distinct forms of the disease have been recognized: the infantile autosomal recessive osteopetrosis, the malignant and the intermediate forms, and autosomal dominant osteopetrosis, type I and type II forms. The autosomal recessive types are the most severe forms with symptoms in very early childhood, whereas the autosomal dominant classes exhibit a heterogeneous trait with milder symptoms, often at later childhood or adulthood.

**Case presentation:**

Case 1 is the 12-year-old daughter (index patient) of an Iraqi-Kurdish family who, at the age of eight years, was diagnosed clinically to have mild autosomal dominant osteopetrosis. Presently, at 12-years old, she has severe complications due to the disease progression. In addition, the same family previously experienced the death of a female child in her late childhood. The deceased child had been misdiagnosed, at that time, with thalassemia major. In this report, we extended our investigation to identify the type of the inheritance patterns of osteopetrosis using molecular techniques, because consanguineous marriages exist within the family history. We have detected one heterozygous mutation in exon 15 of the *Chloride Channel 7* gene in the index patient (Case 1), whereas other mutations were not detected in the associated genes *TCIRG1, OSTM1*, *RANK, and RANKL*. The missense mutation (CGG>TGG) located in exon 15 (c.1225C>T) of the *Chloride Channel 7* gene changed the amino acid position 409 from arginine to tryptophan (p.R409W, c.1225C>T).

Case 2 is the 16-year-old son (brother of the index patient) of the same family who was diagnosed clinically with mild autosomal dominant osteopetrosis. We have identified the same heterozygous mutation in exon 15 of the *Chloride channel 7* gene in this patient (Case 2). The missense mutation (CGG>TGG) located in exon 15 (c.1225C>T) of the *Chloride channel 7* gene changed the amino acid position 409 from arginine to tryptophan (p.R409W, c.1225C>T).

In addition to the clinical diagnosis of both cases, the missense mutation we identified in one allele of the *Chloride channel 7* gene could be linked to autosomal dominant osteopetrosis-II because the symptoms appear in late childhood or adolescence.

**Conclusion:**

In this family, the molecular diagnosis was confirmed after identification of the same mutation in the older son (sibling). Furthermore, we detected that the father and his brother (the uncle) are carriers of the same mutation, whereas the mother and her sister (the aunt) do not carry any mutation of the *Chloride channel 7* gene. Thus, the disease penetrance is at least 60% in the family. The mother and father are cousins and a further consanguineous marriage between the aunt and the uncle is not recommended because the dominant allele of the *Chloride channel 7* gene will be transferred to the progeny. However, a similar risk is also expected following a marriage between the uncle and an unrelated woman. The p.R409W mutation in the *Chloride channel 7* gene has not yet been described in the literature and it possibly has a dominant-negative impact on the protein.

## Introduction

Osteopetrosis (OP) is a clinically and genetically heterogeneous group of diseases characterized by a symmetrical increase in bone density [1,2]. The sclerosis of bone is due to the absence of or to a defect in osteoclast bone resorption. The overall incidence of these conditions, dependent on the disease forms, varies from 1:20,000 to 1:250,000 [1-3].

The severity of the presentation of OP in infancy varies from asymptomatic to fatal and can be grouped into autosomal recessive (ARO) and autosomal dominant (ADO) [1-3]. The severest forms tend to have autosomal recessive inheritance, whereas the mildest forms are inherited in an autosomal dominant manner [3,4]. The malignant and intermediate form of ARO is a life-threatening condition and the disease is manifested in infancy and early childhood [1,3]. The benign ADO typically has an onset in late childhood or adolescence [1,3] and can be subdivided into three subclasses: benign type I (ADO-I), benign type II (ADO-II), and benign type III (ADO-III) [1,5,6]. ADO-I is generally very mild with a diffuse sclerosis without alterations in the bone turnover. Genetic mutations of *Low-density lipoprotein receptor-related protein 5* gene were identified to be responsible for ADO-I [7,8], thus causing an increased bone formation ‘high bone density’ rather than a decreased bone resorption [7,8]. This phenotype is not associated with an increased fracture rate and is reported to be fully penetrant [9], whereas ADO-II has an extremely heterogeneous course ranging from an asymptomatic to a severe phenotype. ADO-II is the most common form; it is characterized by thickness of the vertebral end plates (sandwich vertebrae or rugger jersey spine) and the bone within the bone, and is most commonly noted in the pelvis, vertebrae and at the ends of long bones. ADO-II is associated with diffuse pain, hematological and neural failure, osteomyelitis and frequent pathological fractures. Early death in patients with ADO-II is rare, but some patients can experience a very poor quality of life [10]. In 1904, Albers-Schönberg was the first to describe a case of OP [11], which is an identical OP phenotype to ADO-II symptoms. ADO-III or centrifugal OP has been described in a Vietnamese family; this type is predominantly characterized by sclerosis of the distal appendicular skeleton and the skull, but with minor involvement of the axial skeleton [12].

The families of Chloride channel (CLC) proteins have received considerable attention, often with surprising developments, over the past five years [13], and their physiological roles are impressively illustrated by various inherited human diseases and knockout mouse models [14]. The *Chloride channel 7* (*CLCN7*) gene is a member of the mammalian *CLC* gene family [14]. In osteoclasts, the *Chloride channel 7* (CLC7) protein resides in the late endocytotic–lysosomal pathway of the ruffled membrane borders and is involved in the acidification of the resorption lacunae. The physiological function of the CLC7 protein was unclear until Kornak showed that the disruption of the *CLCN7* gene in mice causes severe OP, and that the CLC7 protein played an essential role in the acidification of the extracellular resorption lacunae, which is very important for osteoclast-mediated resorption of mineralized bone [15]. In humans, mutations in the *CLCN7* gene give rise to the complete spectrum of OP phenotypes, ranging from malignant and intermediate forms of ARO to ADO-II and even asymptomatic ADO-II cases [16-21]. Indeed, the complete clinical and molecular analysis of several patients found novel mutations, including missense, frameshift, nonsense, deletion and splice-site defects [20-22].

The first successful bone marrow (BM) transplantation for OP patients was in 1977 [23,24]. At that time, the biological origin of osteoclasts and osteoblasts was unknown. The OP patients were successfully treated by bone BM transplantation, from the availability of compatible donors of osteoclasts and a mixture of hematopoietic lineages. This was later confirmed experimentally [25]. BM transplantation is a drastic treatment and it is applied only to children severely affected by OP. If it is successful, it will save the life of a child who would otherwise die from the disease progression, which results in growth retardation and neurological defects. Development of hematopoietic stem cell transplantation (HSCT) is a promising treatment for this disease due to the elimination of rejection risks [26]. In the future, an alternative approach that holds the potential for the permanent cure of OP is *in utero* prenatal HSTC (for review see [27]).

## Case presentation

### Case 1

A Kurdish family in Sulaimaniyah city (Kurdistan Region, Iraq) has been identified with a history of OP in siblings. Currently, the family has two children: one affected 12-year-old girl (Case 1), and a 16-year-old boy (Case 2) with milder symptoms of OP. The index patient (Case 1), already diagnosed at the age of eight years with a mild OP phenotype, has now developed a severe form of OP. She is currently reaching the end-stage due to the disease progression: she has anemia, diffuse cutaneous ecchymosis with gum bleeding and recurrent epistaxis, and chest infections. All these clinical features are due to the reduction of BM function and BM cavity. Moreover, she complains of right-sided conductive deafness and a severe reduction in visual acuity due to compression on the cranial nerves. In addition, the family also has a previous history of a daughter with secondary anemia, bleeding tendency, and splenomegaly in her late childhood. She was wrongly diagnosed with thalassemia major at that time prior to her death. Therefore, we decided to screen for the possible mutated gene(s) often associated with OP and determine inheritance pattern for the disease development. We prepared genomic deoxyribonucleic acid (DNA) from peripheral blood samples obtained from the whole family, consisting of father, mother, son, the surviving daughter (Case 1) together with a paternal uncle and a maternal aunt. The peripheral blood samples were preserved in ethylenediaminetetraacetic acid, then the genomic DNA was extracted using the Qiagen Genome DNA kit.

**Figure 1:**
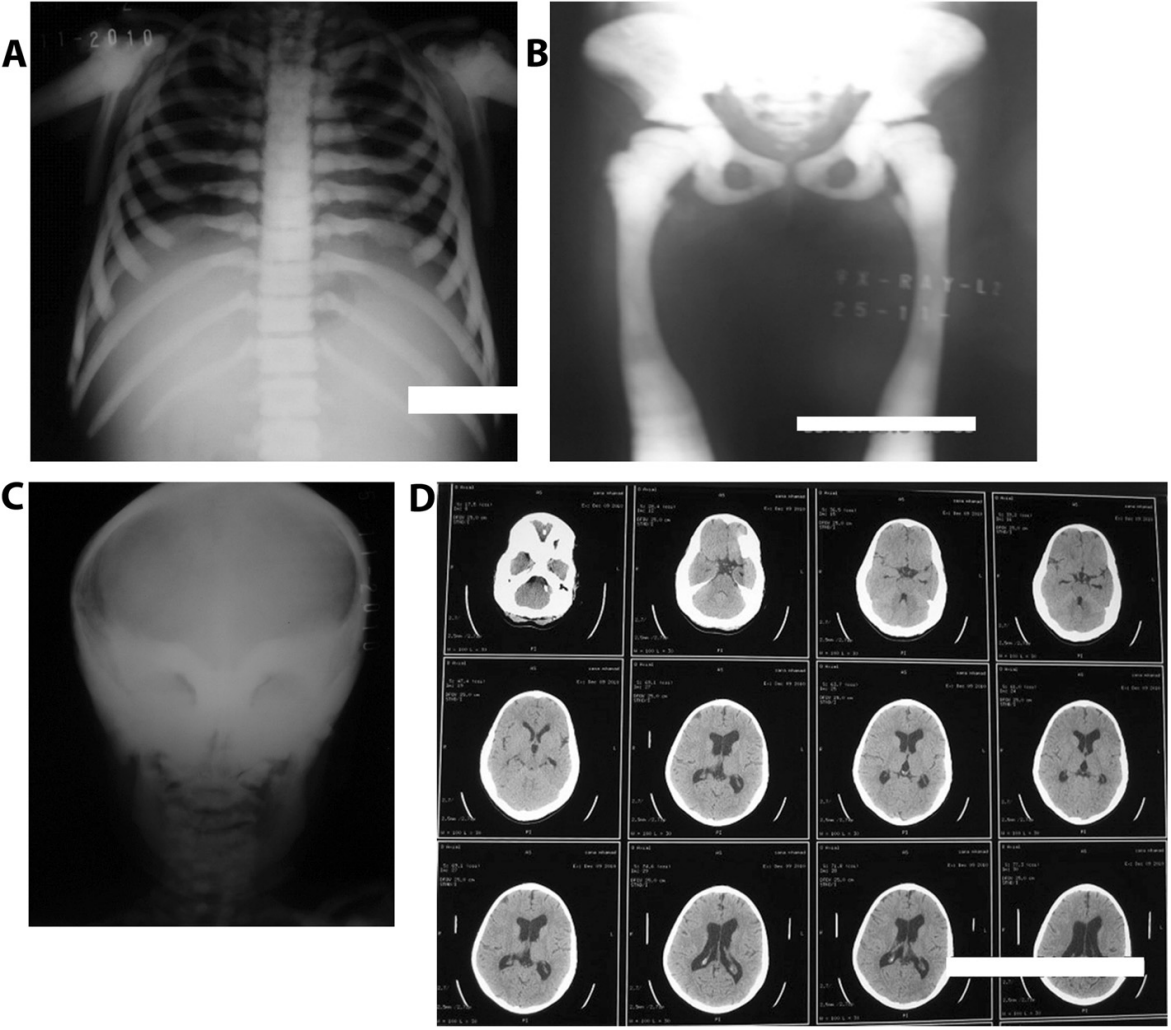
**X-rays and computed tomography scan image of the patient showing bone thickening.** Radiograph shows typical (**A**) vertebral endplate thickening (sandwich vertebrae sign) of the rib and vertebrae, (**B**) diffuse thickening of pelvic and lower limb bones, and (**C**) homogenous sclerotic skull bones. (**D**) A computed tomography scan image of the patient’s brain showing an increased thickened bone density, homogenously sclerotic skull bones encroaching on both optic foramens, and mild dilatation of the lateral and third ventricle with normal fourth ventricle.

The index patient (Case 1) often complained of back pains. Skeletal radiography (Figure 1A-C) showed a diffuse increase in bone density with evidence of a sandwich appearance in the vertebrae and iliac wings (bone-in-bone). The computed tomography (CT) scan (Figure 1D) showed increased thickened bone density, homogenously sclerotic skull bones, encroaching on both optic foramens, and mild dilatation of lateral and third ventricle with normal fourth ventricle.

### Case 2

The same Kurdish family in Sulaimaniyah city (Kurdistan Region, Iraq) has a 16-year-old boy (Case 2) with milder symptoms of OP. The milder grade patient (Case 2) has generally been in good health, but has reported a decline in his visual acuity over the past two years. A clinical laboratory diagnosis was determined for the core family as summarized in Table 1. The results of the laboratory tests clearly show that hemoglobin (Hb), white blood cells (WBCs) and platelet cells are most affected in the index patient (Case 1) in comparison to the parents (Table 1). Other clinical observations show declined organ functions in the index patient compared with normal healthy individuals (Table 2). It is worth mentioning that the reference values for these parameters are not age dependent. Therefore, the data comparison between patients (Case 1 and Case 2) and healthy parents is valid.

**Table 1:** The results of laboratory tests for core family members: the daughter with severe symptoms (Case 1), the son with mild-form symptoms (Case 2) and the parents

**Test**	**Case 1**	**Case 2**	**Mother**	**Father**	**NV**
**Hemoglobin (g/dL)**	**(8)**	13	12	14	12–16
**White blood cell (1000 × cell/dL)**	**(3.5)**	9.0	6.0	8.5	4.0–11.0
**Platelet (1000 × cell/dL)**	**(20)**	240	210	200	150–400
**Blood sugar (mg/dL)**	82	88	86	282	74–110
**Blood urea (mg/dL)**	26	28	22	25	14–50
**Serum creatinine (mg/dL)**	1.1	1.0	1.1	1.3	0.2–1.2
**Serum calcium (mg/dL)**	8.1	9.5	8.2	9.2	8.1–10.4
**Serum phosphorous (ng/dL)**	4.0	3.0	2.8	2.7	2.5–5.0
**Serum alkaline phosphate (IU/L)**	261	515	284	218	100–320
**Serum alanine transaminase (IU/L)**	17	44	35	36	<45
**Serum aspartate transaminase (IU/L)**	30	45	40	38	<35
**Total serum bilirubin (mg/dL)**	1.0	0.4	0.4	0.7	0.2–1.2
**Parathyroid hormone (pg/mL)**	35	32	16	25	10–65
**Serum lactate dehydrogenase (IU/mL)**	416	246	200	206	150–500

*NV*, the range of normal values in healthy individuals, the parents. The bold numbers in parenthesis under Case 1 represent values outside of the normal reference range.

**Table 2:** Other clinical manifestations of the children (Case 1 and Case 2) and healthy parents

**Test**	**Case 1**	**Case 2**	**Mother**	**Father**
**Electroencephalography**	N	N	N	N
**Audiology**	Right otitis media	N	N	N
**Ophthalmology**	Bilateral optic atrophy	Visual acuity = 6 out of 36	N	N
**Spleen**	19cm enlarged	N	N	N
**Liver**	17cm enlarged	N	N	N
**Height**	117cm (<3rd centile)	N	N	N
**Weight**	23kg (<3rd centile)	N	N	N

*N*, Normal state.

## Discussion

The scleroses of bone in patients are often asymptomatic at an early stage and the precise diagnosis is clinical and largely depends on the radiographic appearance of the skeleton. The radiographic appearance exhibits a diffused sclerosis in skull, spine, pelvis and appendicular bones, and a funnel-like appearance and characteristic lucent bands at the metaphysis of the long bones that result from defects of bone remodeling [3]. Other features are also observed, such as bone-in-bone appearance, and focal sclerosis of the skull base, pelvis and vertebral end plates [3]. In other cases the diagnosis may be reached by chance [28]. In the absence of typical radiographic findings, raised creatine kinase BB isoenzyme and tartrate-resistant acid phosphatase (TRAP) can be helpful in making a diagnosis of ADO [29-31]. However, by systematic investigations, in our case, we have diagnosed the patients’ manifestations as related to ADO-II. Our diagnosis was based on clinical symptoms, radiographic appearance and laboratory analysis. The laboratory data (Table 1) showed normal range of serum sugar, urea, creatinine, phosphorus, calcium, bilirubin, alanine transaminase, aspartate transaminase, lactate dehydrogenase and parathyroid hormone for all the family members, except a slight elevation in serum alkaline phosphatase for the brother (Case 2) who has milder symptoms (Table 1). However, we did not analyze creatine kinase BB isoenzyme and TRAP because of the absence of these assays in our locality. The index patient (Case 1) showed lower values for Hb and WBC, and her platelet count was diminished by a factor of 10 (Table 1). Therefore, the index patient (Case 1) showed anemia and thrombocytopenia, whereas the blood examination was normal for other family members (Table 1).

Next, we performed genetic analysis for the whole family, including an aunt and an uncle, in order to link the disease-type with a possible gene(s) mutation and to find out the origin of the inherited condition. We wanted to address possible risks for the development of the disease in children born within the family in the future because the parents are cousins, and because there is the possibility of other consanguineous marriages involving the aunt and the uncle. With these notations, we decided to help the family to perform genetic counseling in this particular case.

**Figure 2:**
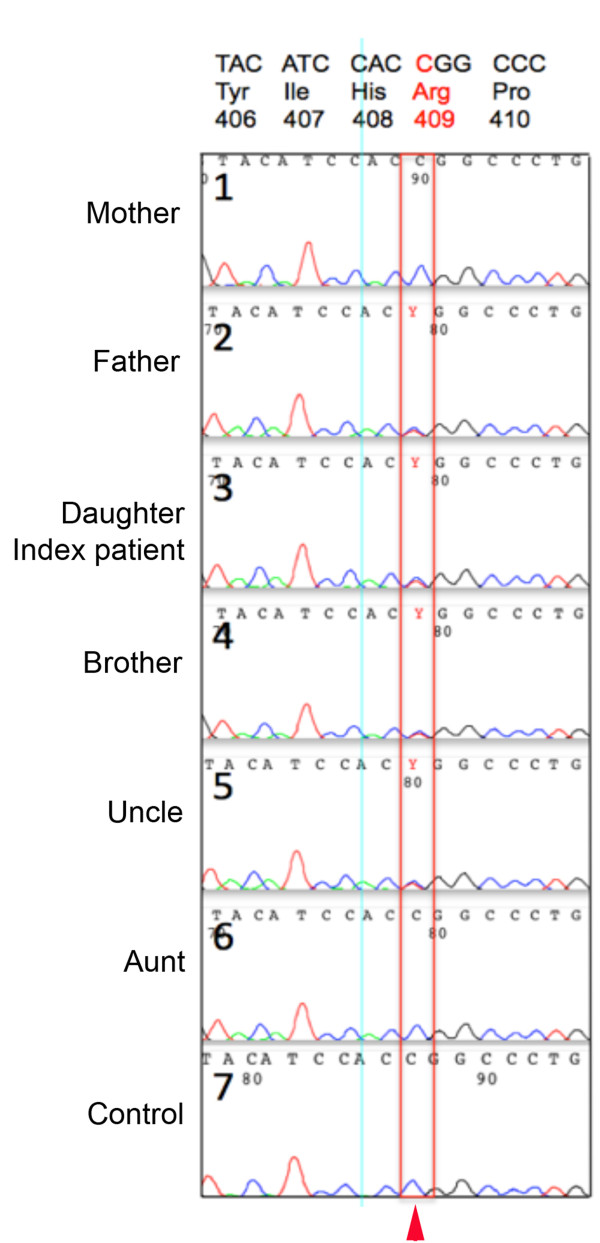
**Electropherogram results of *CLCN7 *exon 15 sequencing derived from a family with a history of autosomal dominant osteopetrosis-II and a control individual.** The c.1225C>T position is indicated. Codon CGG is changed to TGG (p.Arg409Trp).

To this end, we performed systematic sequencing of the genes most commonly associated with OP: *TCIRG1, CLCN7*, *OSTM1*, *RANK* and *RANKL*. The six members of the family, including the parents of the minors, gave written informed consent to participate in this analysis. From the genomic DNA, the coding regions of the above five genes were amplified using the polymerase chain reaction (PCR) method. Complete DNA sequencing on each PCR product was performed and a missense mutation was found at exon 15 of the *CLCN7* gene (Figure 2). In the index patient, sequencing of the other associated genes revealed coding sequences for wild-type protein. Therefore, other family members were exclusively screened for mutation at exon 15 of the *CLCN7* gene (Figure 2).

When the whole coding regions of the *CLCN7* gene were analyzed for the index patient (daughter), a unique heterozygous missense mutation (CGG>TGG) in exon 15 (c.1225C>T) was detected (Figure 2), which changes the amino acid position 409 from arginine to tryptophan of the CLC7 protein (p.R409W). Although the mother and the aunt displayed a wild-type (p.Arg409) amino acid for the CLC7 protein, all the other family members had the heterozygous mutation p.Arg409Trp (Figure 2 and Table 3). The missense mutation (p.Arg409Trp) found in this work is unique and has not yet been described in the published literature. It has previously been found that the heterozygous mutation in the *CLCN7* gene is linked to benign ADO (ADO-II) symptoms [20,21], because mild symptoms appear in early childhood and worsen over time with life-threatening complications. These features have been historically experienced within the family: one child died at age 10 years, another child (index patient) has ADO-II progression at the age of 12 years, whereas their 16-year-old brother had no obvious symptoms at early childhood but clinical features have now started to appear in relation to ADO-II. Our genetic findings of the mutation *CLCN7* gene confirm that the brother is also at risk of developing ADO-II complications.

**Table 3:** **Unique heterozygous mutation in the *CLCN7 *** **gene found in the family**

**Samples**	**Family member**	**Mutation status of the** ***CLCN7*** **gene C1255T (R409W)**
**1**	Mother	Wild type
**2**	Father	Heterozygous
**3**	Daughter (index patient, Case 1)	Heterozygous
**4**	Brother (mild carrier, Case 2)	Heterozygous
**5**	Uncle	Heterozygous
**6**	Aunt	Wild type
**7**	Control	Wild type

**Figure 3:**
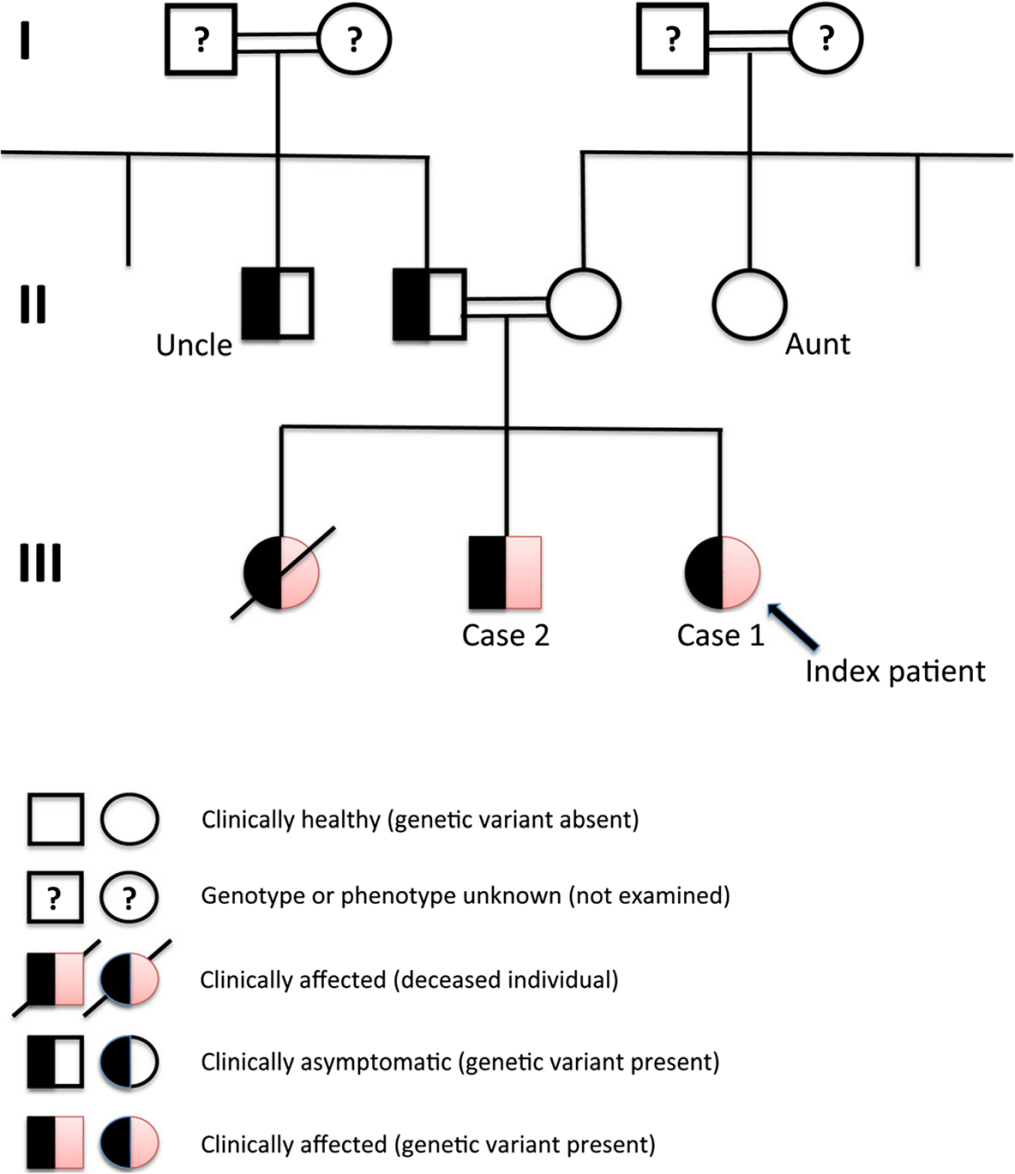
**Family pedigree analysis, in which three individuals (children) are or have been affected by autosomal dominant osteopetrosis-II, while the father and the uncle are carriers.** One of the affected children has died (girl at age 11 years), another is a mild carrier (Case 2, a boy at age 16 years) and the index patient has severe symptoms (Case 1, a girl at age 12 years).

Molecular and clinical findings have shown that various mutations in the *CLCN7* gene lead to the development of various types of OP, from asymptomatic to severe forms [15,17,20,21,32]. Mice with a disruption of the corresponding gene *CLCN7* showed severe OP, retinal degeneration, and they only live for between five and seven weeks [33]. In humans it was also noted that homozygous mutation in the *CLCN7* gene causes malignant infantile ARO [15], whereas patients heterozygous for dominant-negative mutations have a less severe form of ADO-II [32]. It is known that ADO-II symptoms are not homogenous and have wide clinical manifestations from asymptomatic to severe [31,34]. Large scale genotype–phenotype correlation analysis of *CLCN7*-dependent ARO cases and ADO-II cases probably does not support the haploinsufficiency mechanism, rather the hypothesis has to be validated experimentally with biochemical assays [20]. This suggests to us that either the father or the uncle might develop ADO-II symptoms in late adulthood, or they might live a normal life without any obvious ADO-II phenotype. The genetic findings in this work allows us to perform pedigree analysis, indicating the origin of inheritance to be common for the father and uncle (Figure 3).

## Conclusions

In this study, we report a case of a Kurdish family affected by an OP phenotype with broad severity, and diagnosed using complete clinical management, laboratory analysis, and treatment in our locality. We have identified a novel missense mutation (p.R409W) in exon 15 of the *CLCN7* gene as responsible for ADO-II in the 12-year-old index patient and her 16-year old brother; their father is a seemingly healthy carrier of the mutation. Our results show that the uncle (father’s brother) is also a carrier indicating that another possible consanguineous marriage between the aunt (mother’s sister) and the uncle is not recommended. We believe that it is very probable that their progeny would develop ADO-II because they will carry an inherited mutated allele of the *CLCN7* gene. Although unidentified genetic variants might exist, a marriage between the uncle-carrier and an unrelated woman could also develop risks similar to those of his marriage to the non-carrier aunt. However, the complete genotype–phenotype correlation remains obscure, even though one allele of the *CLCN7* gene was found to be mutated. Discovery of a specific biochemical assay marker is required to quantify the actual CLC activity of the CLC7 protein that might support diagnosis more precisely to correlate the CLC function to the disease forms.

## Consent

Written informed consent was obtained from the parents to report the case together with any accompanying images. A scanned-original copy of the written consent is available for review by the Editor-in-Chief of this journal.

## Abbreviations

ADO: Autosomal dominant osteopetrosis; ARO: Autosomal recessive osteopetrosis; BM: Bone marrow; CLC: Chloride channel; CLC7: Chloride channel 7 protein; *CLCN7*: *Chloride channel 7* gene; CT: Computed tomography; Hb: Hemoglobin; HSTC: Hematopoietic stem cell transplantation; OP: Osteopetrosis; PCR: Polymerase chain reaction; TRAP: Tartrate-resistant acid phosphatase; WBC: White blood cell.

## Competing interests

The authors declare that they have no competing financial interests.

## Authors’ contributions

BMR performed the DNA preparations and contributed in writing. NGR performed most of the clinical diagnoses, treatment, and follow-up of the patient and the family. BFN designed, restructured and coordinated the project and wrote the manuscript, including editing, revising and submitting. All the screening DNA-sequencing of the five genes associated with ADO-II were done at the Molecular Diagnosis Laboratories of Ulm University, Germany, by AS and GL. All the participants of this work have read and approved the final manuscript.
